# Helsinki by nature: The Nature Step to Respiratory Health

**DOI:** 10.1186/s13601-019-0295-2

**Published:** 2019-10-30

**Authors:** Tari Haahtela, Leena von Hertzen, Josep M. Anto, Chunxue Bai, Abay Baigenzhin, Eric D. Bateman, Digambar Behera, Kazi Bennoor, Paulo Camargos, Niels Chavannes, Jaime Correia de Sousa, Alvaro Cruz, Maria Do Céu Teixeira, Marina Erhola, Eeva Furman, Bilun Gemicioğlu, Sandra Gonzalez Diaz, Peter W. Hellings, Pekka Jousilahti, Nikolai Khaltaev, Vitezslav Kolek, Piotr Kuna, Stefania La Grutta, Le Thi Tuyet Lan, Tamaz Maglakelidze, Mohamed R. Masjedi, Florin Mihaltan, Yousser Mohammad, Elizabete Nunes, Arvid Nyberg, Jorge Quel, Jose Rosado-Pinto, Hironori Sagara, Boleslaw Samolinski, Dean Schraufnagel, Talant Sooronbaev, Mohamed Tag Eldin, Teresa To, Arunas Valiulis, Cherian Varghese, Tuula Vasankari, Giovanni Viegi, Tonya Winders, Anahi Yañez, Arzu Yorgancioğlu, Osman Yusuf, Jean Bousquet, Nils E. Billo

**Affiliations:** 1 Skin and Allergy Hospital, Helsinki University Hospital, University of Helsinki, Helsinki, Finland; 2 Department of Dermatology, Allergology and Venereology, Helsinki University Hospital, Helsinki, Finland; 3 ISGlobAL, Centre for Research in Environmental Epidemiology (CREAL), Barcelona, Spain; 4 Zhongshan Hospital, Fudan University, Shanghai Respiratory Research Institute, Shanghai, China; 5 EuroAsian Respiratory Society, Astana City, Kazakhstan; 6 Department of Medicine, University of Cape Town, Cape Town, South Africa; 7 Dept. of Pulmonary Medicine, Postgraduate Institute of Medical Education & Research, Chandigarh, India; 8 Department of Respiratory Medicine, National Institute of Diseases of the Chest and Hospital, Dhaka, Bangladesh; 9 Department of Pediatrics, Medical School, Federal University of Minas Gerais, Belo Horizonte, Brazil; 10 Department of Public Health and Primary Care, Leiden University Medical Center, Leiden, The Netherlands; 11 Life and Health Sciences Research Institute, ICVS, School of Medicine, University of Minho, Braga, Portugal; 12 ProAR – Nucleo de Excelencia em Asma, Federal University of Bahia, Vitória Da Conquista, Brazil; 13 Hospital Dr Agostinho Neto, Praia, Cabo Verde; 14 National Institute for Health and Welfare (THL), Helsinki, Finland; 15 Environmental Policy Centre, Finnish Environment Institute, Helsinki, Finland; 16 Department of Pulmonary Diseases, Cerrahpasa Faculty of Medicine, Istanbul University-Cerrahpasa, Istanbul, Turkey; 17 Hospital Universitario y Facultad de Medicina, Monterrey Nuevo Leon, Mexico; 18 Laboratory of Clinical Immunology, Department of Microbiology and Immunology, KU Leuven, Louvain, Belgium; 19 Global Alliance Against Chronic Respiratory Diseases (GARD), Geneva, Switzerland; 20 Department of Respiratory Diseases and Tuberculosis, University Hospital Olomouc, Olomouc, Czech Republic; 21 Division of Internal Medicine, Asthma and Allergy, Barlicki University Hospital, Medical University of Lodz, Lodz, Poland; 22 Istituto per la Ricerca e l’Innovazione Biomedica (IRIB), Consiglio Nazionale delle Ricerche (CNR), Palermo, Italy; 23 Respiratory Care Center, University Medical Center, Ho Chi Minh City, Vietnam; 24 Pulmonology Department, Ivane Javakhishvili Tbilisi State University, Chapidze Emergency Cardiology Center, Tbilisi, Georgia; 25 Shahid Beheshti University of Medical Sciences, Tehran, Iran; 26 National Institute of Pneumology M. Nasta, Bucharest, Romania; 27 National Center for Research in Chronic Respiratory Diseases, Tishreen University School of Medicine, Latakia, Syria; 28 Pulmonology Department, Maputo Central Hospital, Maputo, Mozambique; 29 FILHA, Finnish Lung Health Association, Helsinki, Finland; 30 Hispanic American Allergy Asthma & Immunology Association, Marina Del Rey, California USA; 31 Immunoallergology Department, Hospital da Luz Lisboa, Lisbon, Portugal; 32 Division of Allergology & Respiratory Medicine, Showa University School of Medicine, Tokyo, Japan; 33 Department of Prevention of Environmental Hazards and Allergology, Medical University of Warsaw, Warsaw, Poland; 34 Department of Medicine, University of Illinois at Chicago, Chicago, USA; 35 Kyrgyzstan National Centre of Cardiology and Internal Medicine, Euro-Asian Respiratory Society, Bishkek, Kyrgyzstan; 36 Department of Thoracic Diseases, Ain Shams Faculty of Medicine, Abbassia, Cairo, Egypt; 37 The Hospital for Sick Children, Research Institute and Della Lana School of Public Health, University of Toronto, Toronto, ON Canada; 38 Clinic of Children’s Diseases, Institute of Clinical Medicine, and Department of Public Health, Institute of Health Sciences, Vilnius University, Vilnius, Lithuania; 39 Management of NCDs, WHO, Geneva, Switzerland; 40 Istituto di Fisiologia Clinica CNR, Pisa, Italy; 41 Allergy & Asthma Network, Vienna, VA USA; 42 Global Allergy & Asthma Patient Platform, Vienna, Austria; 43 Investigaciones en Alergia y Enfermedades Respiratorias (INAER), Buenos Aires, Argentina; 44 Department of Pulmonary Diseases, Faculty of Medicine, Celal Bayar University, Manisa, Turkey; 45 The Allergy and Asthma Institute, Islamabad, Pakistan; 46 MACVIA-France, Fondation Partenariale FMC VIA-LR, CHRU Arnaud de Villeneuve, Montpellier, France; 47 Global Alliance Against Respiratory Diseases (GARD), Helsinki, Finland

**Keywords:** Nature, Biodiversity, Immune regulation, Lifestyle, Respiratory diseases, Environment, Planetary health, CRDs, NCDs, SDGs

## Abstract

**Background:**

*The Nature Step to Respiratory Health* was the overarching theme of the 12th General Meeting of the Global Alliance against Chronic Respiratory Diseases (GARD) in Helsinki, August 2018. New approaches are needed to improve respiratory health and reduce premature mortality of chronic diseases by 30% till 2030 (UN Sustainable Development Goals, SDGs). Planetary health is defined as the health of human civilization and the state of the natural systems on which it depends. Planetary health and human health are interconnected, and both need to be considered by individuals and governments while addressing several SDGs.

**Results:**

The concept of the Nature Step has evolved from innovative research indicating, how changed lifestyle in urban surroundings reduces contact with biodiverse environments, impoverishes microbiota, affects immune regulation and increases risk of NCDs. The Nature Step calls for strengthening connections to nature. Physical activity in natural environments should be promoted, use of fresh vegetables, fruits and water increased, and consumption of sugary drinks, tobacco and alcohol restricted. Nature relatedness should be part of everyday life and especially emphasized in the care of children and the elderly. Taking “nature” to modern cities in a controlled way is possible but a challenge for urban planning, nature conservation, housing, traffic arrangements, energy production, and importantly for supplying and distributing food. Actions against the well-known respiratory risk factors, air pollution and smoking, should be taken simultaneously.

**Conclusions:**

In Finland and elsewhere in Europe, successful programmes have been implemented to reduce the burden of respiratory disorders and other NCDs. Unhealthy behaviour can be changed by well-coordinated actions involving all stakeholders. The growing public health concern caused by NCDs in urban surroundings cannot be solved by health care alone; a multidisciplinary approach is mandatory.

## Background

The 12th General Meeting of the Global Alliance against Chronic Respiratory Diseases (GARD) [1] was hosted by the Finnish Lung Health Association and the National Institute for Health and Welfare in Helsinki 30.8.-1.9.2018. It covered the theme *The Nature Step to Respiratory Health* discussing the influence of nature and natural elements on respiratory health and on NCDs in general. Prevention and management of chronic respiratory diseases need a fresh approach, especially as new data concerning health effects of environment, lifestyle and indigenous microbiota have emerged since previous GARD meetings. The meeting gathered around 100 opinion leaders from all continents and over 30 countries. It featured presentations on hot topics like environmental effects on respiratory health, determinants of immune balance and planetary health.

This paper outlines the recent findings in the area of improving contact with nature as a strategy for respiratory health and suggests action to combat the challenges of the modern world. To change our behaviour, we should first change our minds by adapting new knowledge [2].

The United Nations Sustainable Development Agenda 2030 was formulated by world leaders in 2015 at a historical summit in New York [3]. All countries were called to work on 17 sustainable development goals (SDGs) and end poverty, fight inequalities, tackle climate change and ensure that no one is left behind. These goals highlight that education, health, social protection, tackling climate change and restoring the natural resources of the Earth are important prerequisites to achieve economic balance and end poverty [4]. While the SDGs are not legally binding, governments and populations are urged to take ownership, show commitment, provide the necessary funding and monitor progress towards the goals within the timeframe of 11 years, by the year 2030.

Over the last few decades, premature mortality from non-communicable diseases (NCDs) before age 70 has dropped in almost all countries [5]. However, additional efforts are needed to achieve a further reduction of 30% by 2030. SDG Goal No. 3 on *Good health and wellbeing* promotes healthy living for all and lists a number of important targets to be achieved. For NCDs it aims to reduce premature mortality by one-third through prevention and treatment, and promote mental health. SDG Goal No. 15 on *Life on Land* proposes we take care of our planet. This includes stopping deforestation, land degradation and loss of animal and plant species, i.e. biodiversity loss. Contact with nature educates the human immune system and endorses tolerance against a variety of exposures being thus an essential determinant of health [6]. All the 17 goals are highly interconnected and in many cases progress in one depends on progress in some other goals. For example, biodiversity is a goal the progress of which several other goals depend on [7].

## The concept of Nature Step

### Urbanization and sedentary lifestyle

**Fig. 1:**
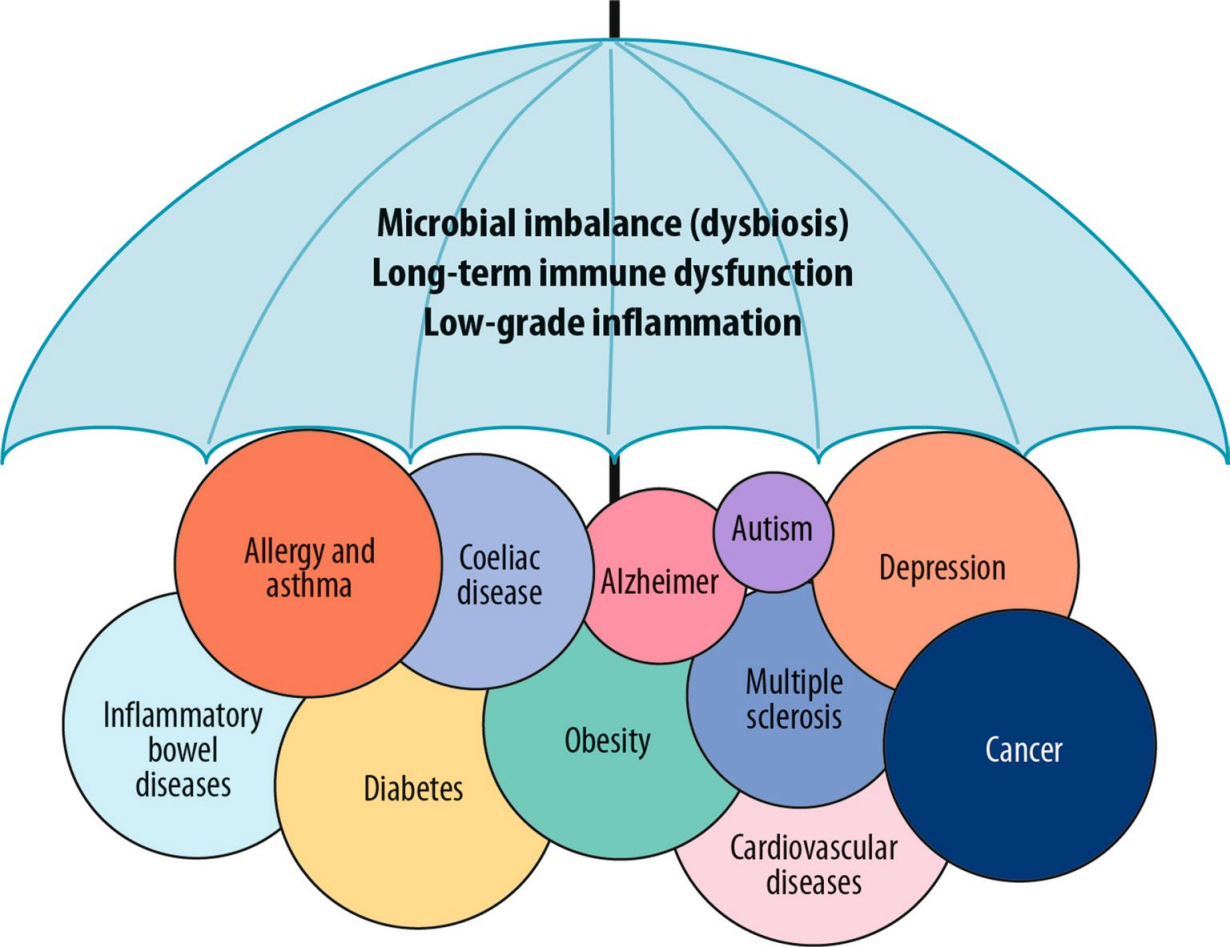
Several non-communicable diseases have been suggested to share the same underlying risk factors such as microbial imbalance, long-term immune dysfunction and low-grade inflammation

The world is urbanizing faster than ever, and the United Nations predicts that 68% of all human populations live in cities by 2050 [8]. At the same time many chronic conditions including respiratory, allergic, autoimmune, metabolic and mental diseases, are on the increase worldwide in urban environments [9, 10]. The human immune system has run into an adaptation crisis not having had time to adjust to the rapidly changing environment and lifestyles. Crucial elements in this context are the environmental as well as our indigenous microbiota [11] (Fig. 1).

Urban living in asphalt-covered environments with little green space may not provide us with the diverse microbial stimulation necessary for the development of a balanced immune function. This is augmented by the use of highly processed food, salty and fatty food, sugary drinks and alcohol and the lack of physical activity. Several chronic diseases mentioned above are linked to alteration in our indigenous microbiota and the disappearance of ancient species from these commensal communities [11].

Other environmental stressors like air pollution, common environmental chemicals, noise and behavioural changes like sedentary lifestyle add to the risks. People in urban settings spend more than 90% of their lives indoors. Sedentary lifestyle has indeed become a serious concern in modern societies [12]. The situation is particularly alarming among children. Recent observations link the lack of green surroundings to mental well-being and depression [13, 14]. The studies of residential surrounding greenness and proximity to green spaces on respiratory and allergic symptoms are not uniform, but the majority shows benefits [15–20]. In a recent study, green areas around school neighbourhoods had an effect even on students´ lung function [21]. Interestingly, this effect was partially mediated by the autonomic nervous system. In a nationally representative cohort of 20,000 subjects in England, at least 120 min weekly contact with natural environment was associated with good health and well-being [22].

Increasing surrounding greenness alone may be ineffective, if nutrition and physical activity remain unchanged. Moreover, green surrounding may be more of a surrogate marker of the lifestyle.

### Biodiversity hypothesis

The 2018 Living Planet Report by the World Wildlife Fund gave a stunning message: “Wildlife populations show continuous decline, on average by 58% between 1970 and 2014 and are likely to reach 67% by the end of the decade” [23]. The human impact is overwhelming. According to the Intergovernmental Science-Policy Platform on Biodiversity and Ecosystem Services (IPBES), 75% of the land surface and 66% of the ocean area are significantly altered [24]. Over 85% of wetlands have been lost.

Biodiversity loss may be the most dangerous megatrend, along with interlinked global warming [25, 26] and air pollution [27]. In 2015, for the first time, the UN recognized biodiversity as an essential determinant of human health [9] and included it in Goal No. 15 of the SDG 2030 Agenda [3]. Biodiversity loss in the wider environment was recognized to reduce diversity in human microbiota, contributing to immune dysfunction and disease. For example, environmental and lifestyle changes may affect microbial diversity of the fetal and infant gut microbiome impacting type I diabetes susceptibility [28].

The species on Earth are interlinked by complex interactions like antagonistic ones involving predation, herbivory and parasitism, or mutualistic ones, such as those involving the pollination of flowers by insects. Moreover, the metaphor hints that the interactions may be complex to the point of being impossible to fully elucidate [29]. Biodiversity can be broadly defined as the variety of life on Earth. It includes the genes in all living cells, populations, species and their communities, the habitats in which they occur, and the ecosystems they comprise [30].

The biodiversity hypothesis proposes simply that biodiversity loss leads to immune dysfunction and disease [31]. Reduced contact of people with natural diverse environments, including microbiota, adversely affects the assembly, composition and quality of human commensal microbiota and may thereby lead to inadequate and unbalanced stimulation of immunoregulatory circuits and ultimately to clinical disease [32–35].

The hypothesis is based on the concept that pathogen recognition receptor signalling and the regulatory network activation are needed throughout life for the balanced development and maintenance of immune regulation [6]. Beneficial effects of microbiota in the farming environment are known as it has been shown that children raised in farms with early life exposure to rich microbiota are protected against allergies and asthma [36–38]. However, the role of environmental microbiota by and large has been less explored and recognized. Increased risk in the farming environment may be massive and sudden exposure to microbes causing allergic alveolitis or long term exposure to pesticides [39].

#### Two layers of biodiversity

We are protected by *two nested layers of biodiversity*, consisting of microbes residing in our bodies and those of the environment we live in [40]. The diversity and composition of the *inner layer* are dependent largely on microbial colonisation from the *outer layer*, a process that depends on our environment and behaviour. Microbes are also transferred vertically, from mother to child. This route of microbial colonization has been discussed in detail elsewhere [41]. After a house move, the microbial community in the new house rapidly converged on the microbial community of the occupants’ former house, suggesting colonization by the family’s microbiota [42]. To preserve our inner biodiversity—which closely interacts with the immune system—we need to preserve the outer biodiversity and change our everyday practices. It is evident but poorly studied that everything we *eat, drink, inhale and touch* affects the composition and function of our microbiota and promotes a cross-talk of human DNA with the environmental metagenome [11, 43, 44].

#### The role of microbes in immune tolerance

During the last decade, human microbiota has become a central issue in health and disease. Microbes hold promise for new strategies of prevention and treatment of many inflammatory conditions [45, 46]. Altogether, several factors have been identified to be involved in poorly developed or broken immune tolerance. These include lack of natural microbial exposure, especially in early and late life, dietary factors, dwelling and its surrounding, lifestyles and the use of antibiotics. Broken tolerance is discussed thoroughly elsewhere and not reiterated here [6].

The number of bacteria in the body is about the same as the number of our own cells [47]. Around 3 million genes are encoded in the genome of our microbiota, compared to around 20,000–23,000 genes of the human genome. The microbiome can be regarded as our *second genome*, to which we have externalized many protective and life supporting functions [48]. The gut microbiome is being attributed an important role in diseases such as obesity, diabetes and metabolic disease [49].

Many urban environments appear to lack elements such as plants and trees necessary for the proper development of tolerance against foreign proteins [50]. People living in densely built urban areas are less exposed to diverse environmental microbiota than people living in more sparsely built areas [51]. A study comparing adolescents in Finnish and more rural Russian Karelia [52] showed that the skin and nasal microbiome of the Finnish and Russian adolescents were quite contrasting and directing immune responses to opposite routes [53]. The environmental microbiota may have profound effects on DNA methylation e.g. of CD14, which is a pattern-recognition receptor for lipopolysaccharides (LPS) and other bacterial wall-derived components [54]. Epigenetic regulation affects innate immune function and guides inflammatory pathways [55].

Cities are built and organized differently, and many have residential areas with a lot of green spaces. Practical actions for greener cities are increasing, and also promoted by United Nations [56]. Also, the GSDR 2019 calls upon fostering urban citizens relationship with nature by promoting green space, urban biodiversity and urban food production [7]. At the same time, the idea of *Smart Cities* with environmental priority is one of the central themes to be funded by the new EU Programme, *Horizon Europe 2021*–*2027* [57].

### Nature Step in practice

The Nature Step is still a hypothesis, but suggests *practical actions* to improve nature relatedness by: (i) strengthening connections with natural environments and increasing physical activity, (ii) increasing use of fresh vegetables and fruits and water, avoiding sugary drinks and consumption of tobacco and alcohol, (iii) linking with natural elements especially in the care of children and the elderly, and (iv) focusing research also on ecosystem services and their health effects to gain evidence to improve practices [58].

According to the World Health Organization (WHO), approximately 1.7 million (2.8%) of deaths worldwide are attributable to low fruit and vegetable consumption [59]. There is convincing evidence that consumption of high-energy foods, such as processed foods containing lot of fats and sugars, promotes obesity compared to low-energy foods such as fruits and vegetables, and even increase all-cause mortality [60]. The possible immunological effect of soil microbes in fresh food is a research priority, as in a recent mouse model study soil exposure modified the gut microbiota and supported immune tolerance [44]. Other research priorities in the field of chronic respiratory and allergic diseases have been outlined in a 2010 GARD publication [61].

At the urban society level, there is no return to the traditional farming life, but it is possible to integrate elements of nature into modern cities in a controlled way and foster the contact of humans and green elements through policy and practice. That is a challenge for policy makers responsible for city planning, housing, traffic arrangements, supplying energy, education, social services and especially for food production and distribution. Healthy behaviour may also be promoted by introducing taxes on unhealthy foods and sugary drinks, tobacco and alcohol. The impact of this approach on respiratory health and other NCDs—and reduction in health care costs—is a research priority.

While many of the points included in the Nature Step approach are part of the best buys strategy of the WHO to reduce the burden from NCDs, better understanding of the mechanisms of *nature connection* leading to interventions is needed to obtain evidence for societal actions. People must become aware that the link with nature is critical for their own health as well as for their communities.

#### The Finnish Programme showing the way

**Fig. 2:**
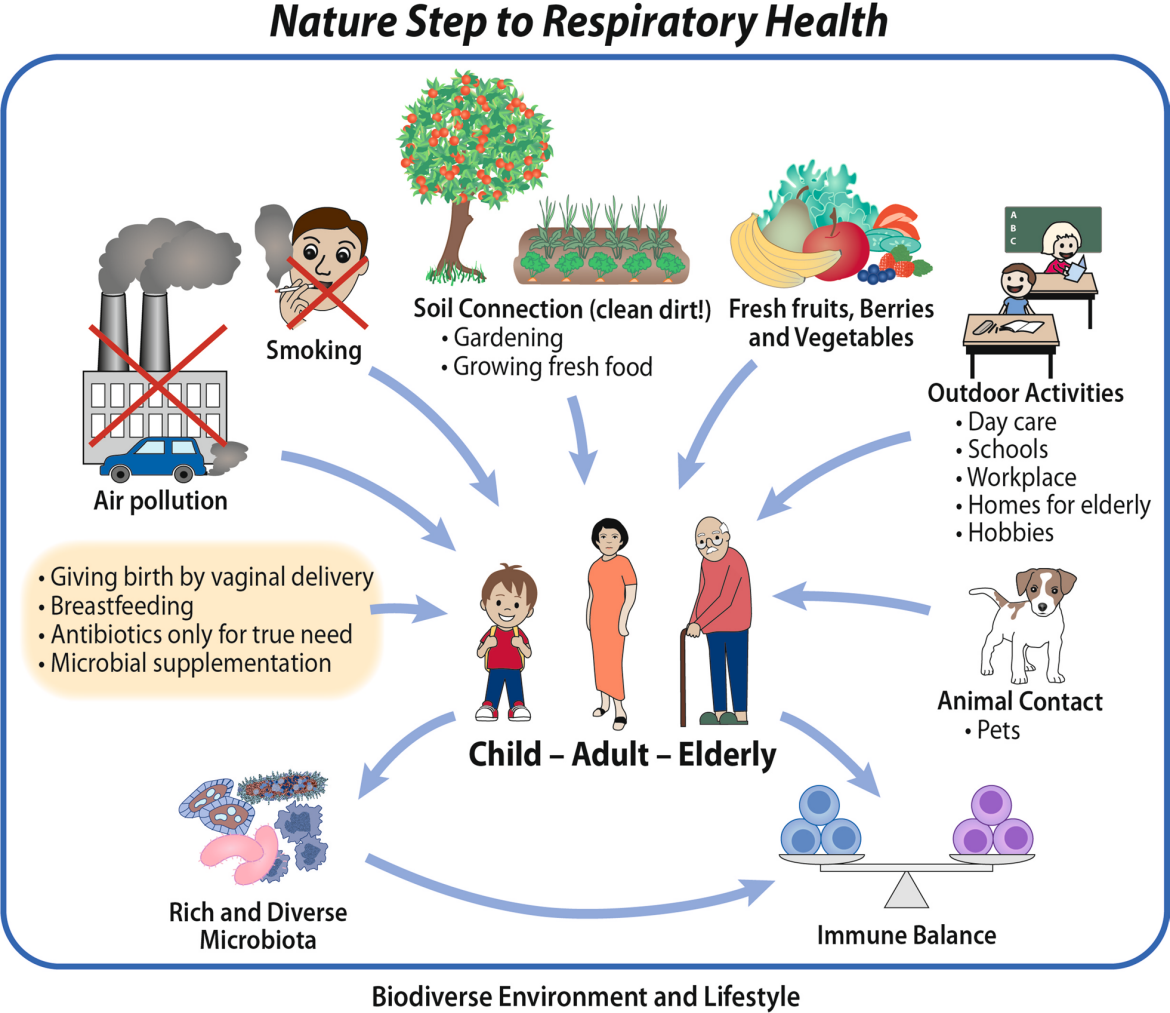
The Nature Step to Respiratory Health

The Finnish Allergy Programme (2008–2018) revisited the allergy and asthma paradigm and led to actions relevant to society and healthcare as a whole [62, 63]. Immune tolerance and allergy health were promoted through a Nature Step in trying to reset the connection between humans and the natural environment, the original home of *Homo sapiens* (Fig. 2). There is some direct evidence indicating that human microbiota can be modulated by nature contact, i.e. by handling soil and plant-based materials [64].

In Finland, the burden of allergy and asthma has started to decline and there is less medicalisation, less allergy diets, and the severity of asthma has decreased. For example, in 2013–2015, the prevalence of use of allergy diets decreased by 43% in day care centres in the Helsinki Capital area [65]. The Finnish disease surveillance system is showing signs that the epidemic is slowing down as the asthma and allergic rhinitis prevalence is levelling off [66]. Experience shows that medical communities and societies can lessen the disability and costs caused by these disorders and improve public health.

Actions taken for allergy and asthma may also show the way to prevent many other NCDs which are on the rise everywhere in urban communities. An educational programme tackling diabetes, obesity and inflammatory bowel diseases, in addition to allergy and asthma, is planned to take off in Finland in 2020 [28, 67]. A Nature Step is also undertaken in day-care, where (i) diet is changed (less meat and more fresh fruits and vegetables), (ii) food waste is minimized, and (iii) connection to natural environments is increased. The project starts in 2019 and is funded by The Finnish Innovation Fund SITRA [68].

The Finnish Allergy Programme (2008–2018) implemented *Nature Step* both for primary and secondary (tertiary) prevention of allergy and asthma by emphasizing nature relatedness. Promoting physical exercise, reducing air pollution and stopping smoking were also central.

## Human and planetary health—the grand challenges

### Planetary health and global warming

According to recent projections, changes in climate will increase in the coming years [69]. Global warming represents a massive threat also to respiratory health by directly promoting or aggravating respiratory diseases, and by increasing exposure to risk factors [70]. Warming increases the exposure to pollen, allergens produced by plants, mould proliferation, ambient air ozone and particulate matter at ground level. The main respiratory concerns are allergic respiratory diseases, asthma, chronic rhinosinusitis (CRS), chronic obstructive pulmonary disease (COPD) and respiratory tract infections. Groups at higher risk of global warming include individuals with existing cardiopulmonary diseases or disadvantaged individuals. Adaptation and mitigation measures are needed.

Climate affects weather, air and water quality, local and national water and food supplies, economics and other critical health determinants. Observational evidence indicates that regional temperature increases affect a diverse set of physical and biological systems in many parts of the world, some of which are of concern for respiratory health. A rapid rise has been observed in the number of hot days, such as the 2003 heat wave resulting in 40,000 excess deaths across Europe, mostly for cardiopulmonary causes [71]. In 2018 another heat wave was experienced in many parts of the world, the effects of which have not been calculated yet. On the other hand, nature based solutions can help humans to adapt to heat waves in cities, e.g. by providing shelter in the form of trees [72].

In 2015, the report of a commission on planetary health created by the Lancet Commission and the Rockefeller Foundation proposed a new way of understanding the relationship between human health and the environment [73]. The view emerged from the realization that humanity is experiencing substantial improvements in life expectancy and health at a time when many ecosystems worldwide are degrading at unprecedented rates. Wealthy populations can use ecosystem services from other locations through access to markets widening health and ecological inequalities. The dependence of health on ecosystems is delayed and complex enough not to be detected with our current paradigms and methods [74, 75]. The current concept of health does not take into account whether health gains are achieved at the cost of eroding the Earth’s underpinning natural systems. To reconcile human health with the restauration of planet’s natural resources, *Planetary Health* is seen as the highest attainable standard of health and wellbeing; i.e. taking into account Earth’s natural systems limits within which humanity can flourish [73].

### Air pollution

Ambient air pollution is a heavy burden in many industrialized and developing countries, especially in urbanized areas where it contributes to increased morbidity and mortality [76]. More than 90% of air pollution related deaths occur in low and middle-income countries According to WHO estimates, outdoor air pollution caused about 4.2 million deaths in 2016 and indoor air pollution from cooking with polluting technologies 3.8 million deaths in the same year [77]. As reported by the *Lancet* Commission on pollution and health, the global estimated annual deaths due to pollution risk factors ranges between 8.4 (according to the WHO best estimate) and 9.0 million (Global Burden of Diseases best estimate) [78].

Lungs and the cardiovascular system are affected by exposure to fine and ultrafine particles in polluted air, causing stroke, heart disease, cancer, COPD and respiratory infections such as pneumonia [79]. A joint ERS/ATS policy statement of the adverse effects of air pollution has been recently published [80]. Outdoor air pollution is a risk factor for asthma and COPD emergency visits [81, 82], and sleep apnoea [83].

For allergy and asthma, immune dysfunction poses the main risk, but heavy air pollution also contributes to inflammation and affects immune regulation, e.g. diesel fumes may promote allergic inflammation [84]. Epidemiological studies in Japan showed that the increase in cedar pollinosis was likely linked to Diesel exhaust [85]. Other outdoor air pollutants are associated with increased frequency of asthma exacerbations as well as symptoms that affect quality of life such as cough, wheezing and nasal drainage [86]. Nevertheless, there are situations like in Finland where ambient air pollution is minimal, even in cities, still asthma prevalence is high [87].

### Smoking

Tobacco use is the main risk factor for all major NCDs. It is estimated by WHO that the tobacco epidemic kills more than 7 million people a year. Six million of these deaths are due to direct tobacco use and about 900,000 due to second-hand smoke [88].

For COPD and lung cancer, smoking is overwhelmingly the greatest risk, which is augmented by air pollutants. Global efforts for implementing the Framework Convention on Tobacco Control and using the *WHO MPOWER package* have led to better policies in tobacco control [89, 90]. In many countries, however, additional efforts are needed to achieve the UN Sustainable Goals. Moreover, since use of electronic cigarettes has rapidly escalated among youths and are strongly associated with the subsequent initiation of combustible tobacco products, control strategies at the national level are mandatory [91].

Furthermore, oriental water pipe (Narghile) smoking is alarmingly increasing in Europe and the Americas and becoming a pandemic [92]. The WHO Framework Convention on Tobacco Control Secretariat has worked to establish a network of six knowledge hubs for the tobacco *MPOWER programme* within academic institutions. Water pipe and smokeless tobacco use is one of the six hubs. Each of them specializes in a given area, such as taxation or research and surveillance, and assists parties in their implementation work and disseminating information.

#### Endgame for smoking

Of particular interest are tobacco endgame policies adopted by a few countries including Finland [93, 94]. The goal of the *Tobacco*-*free Finland 2030 Network* is to create a tobacco- and nicotine-free country [95]. In 2010, 23% of Finnish men and 16% of women smoked. Instead of restricting the harmful effects of smoking, the goal of the Tobacco Act aimed to end the consumption of tobacco products in Finland by the year 2030.

In 2012, Finnish sales outlets were prohibited from displaying tobacco products. In 2014, the European Union passed the updated Tobacco Products Directive (2014/40/EC) stipulating that tobacco packaging must include health warnings containing image and text.

In 2015, 16% of Finnish men and 12% of women smoked. In 2016, the updated version of the Tobacco Act came into force. In 2020, the aim is to ban menthol as a flavouring of tobacco products. In 2030, when the objective of the Tobacco Act is hopefully met, less than 5% of the adult population should consume tobacco or nicotine products on a daily basis.

Under the framework of planetary health, fight against smoking and air pollution provides an opportunity to work both to protect human health and restore the natural resource of the Planet. For air pollution there is abundant evidence that active transportation policies can reduce greenhouse gas emissions and improve air quality and physical exercise [96]. In a similar way, eradication of smoking could not only result in unprecedented health benefits but also in reduction of deforestation and land degradation as well as in improvements of biodiversity in large tobacco production areas [97]. Recent studies have shown that vaping is also representing a risk for morbidity and mortality and needs to be urgently regulated [98].

### Future challenges

The epidemic of chronic respiratory diseases and other NCDs is the result of changes in lifestyle including reduced contact to natural environments, tobacco smoking and outdoor/indoor air pollution and unhealthy diets. Indoor life in buildings, reduced physical activity, diets using processed food and excessive meat consumption rather than plant-based diets [99, 100], sugary drinks, tobacco and alcohol [101] contribute to the risk. The relative importance of each of these factors varies between populations and living conditions, but they are all mainly caused by exponential growth of human populations leading to escalating urbanization worldwide.

**Fig. 3:**
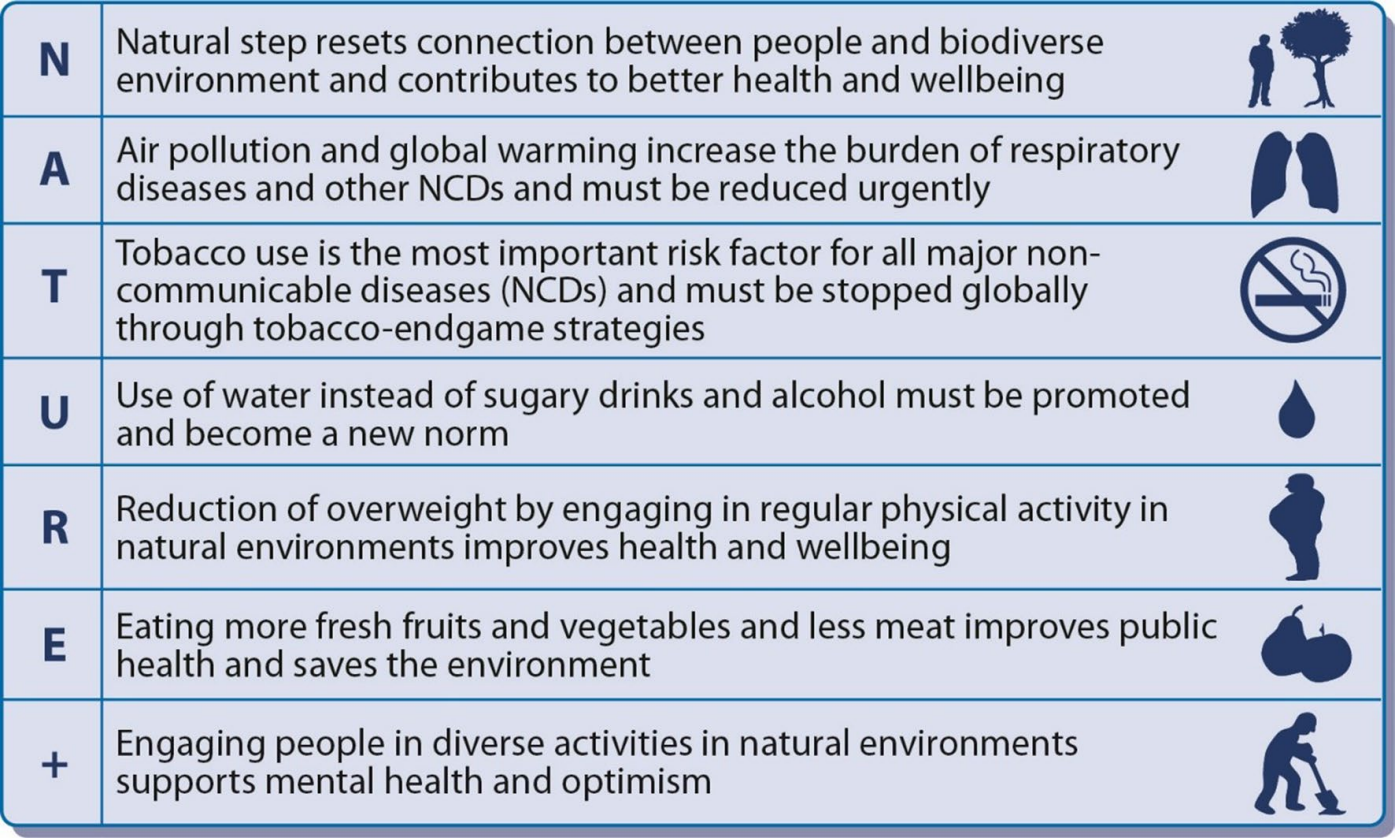
Imperative actions to promote human health and conserve nature

Global, national and local action plans taking into account the local situation need to be constructed and implemented by engaging policy makers, governments, civil society and each individual. This will lead to a better understanding regarding the benefits of positive Steps living in and with Nature (Fig. 3).

Monitoring of the different determinants in urban surrounding and their effects on microbiome and immune regulation is difficult and only little studied. Basic questions remain unanswered. For example, what happens when we pick up a wild berry from the bush to the mouth? What is the microbiota of the berry, how does it transfer to our hands and skin, how to the mouth and gut, and how does it modulate human microbiota and regulate the immune system? If eating wild berries, “superfood”, reduces disease risk, by what mechanism? What is the dynamics of environmental microbiota affecting human microbiota? What is the composition of “healthy” human microbiota and what are the mechanisms of the cross-talk with human cells and gene expression? For example, Sberro et al. found recently thousands of previously unknown small proteins in the human microbiome, which may perform diverse functions including epigenetic modulation [102].

Furthermore, how are human microbiota affected by different diets, antibiotics or chemicals? What are the most important urban/rural microbial determinants influencing risk of NCDs, and what, altogether, is the relative importance of environment/lifestyle factors and hereditary dispositions? Is it possible partly to compensate the “lost nature connection” with artificial microbial supplementation? New information is urgently needed, and we strongly advocate both for controlled and real-life studies.

The logistic regression models to assess air pollution effects should be supplemented by biodiversity information, at least by land use data. Usually, air pollution effects are seen in big cities where biodiversity loss is also at its worst. Their interaction and confounding effects should be investigated in future epidemiological studies. Altogether, the respiratory effects of biodiversity loss and global warming may be enormous but have been insufficiently evaluated [31].

A large proportion of NCDs is preventable and changes in behaviour modify disease severity and outcome. This has been shown in Finland, where several successful public health programmes for chronic respiratory conditions and cardiovascular disease prevention have been implemented [103, 104]. In terms of air pollution, the Vilnius Declaration originating from a meeting by the European Forum for Research and Education in Allergies and Airway Diseases (EUFOREA) in March 2018 proposes several urgent actions to mitigate air pollution [105]. More recently, on September 2018, the United Nations High-level Meeting on Non-communicable Diseases has issued a declaration [106], in which the role of air pollution is outlined as a major risk factor to be combatted. Following this path, WHO has organized the First Global Conference on Air Pollution and Health, October 2018 [107].

While there is plenty of evidence that NCDs are preventable through policy changes such as tax increases for tobacco, unhealthy foods and drinks and individual behaviour change, implementing these measures is challenging as there is lack of funding for prevention programmes and opposition from industries with vested interests.

All governments need to address the health effects of major environmental threats on a regular basis to prompt timely and concrete actions. Indeed, the WHO 12th recommendation from the Declaration of the Health of People, Health of Planet and Our Responsibility: Climate Change, Air Pollution and Health Workshop 2017 states “Promote an alliance with society that brings together scientists, policy makers, healthcare providers, faith/spiritual leaders, communities, and foundations to foster the societal transformation necessary to achieve our goals in the spirit of Pope Francis’s encyclical *Laudato si*” [108].

The present paper is of importance to sustain Planetary Health and should be embedded in next-generation care pathways for respiratory diseases [109] for a change management strategy concerning CRDs [110]. On December 3–4, 2019, a high-level meeting will be organized during the Finnish Presidency of the EU Council to discuss the impact between Planetary and Human health. A focus will be made concerning biodiversity and the digital transformation of health.

## Conclusive remark

New research suggests that reducing harmful exposures and strengthening immune tolerance could be promoted through a Nature Step, resetting the connection between humans and nature. This is also an imperative for nature conservation and safeguarding a peaceful planet.

The most urgent challenge is for the quickly urbanising developing countries as their NCDs epidemic is quite recent and worsening, and effective strategies for prevention and treatment have not been implemented. The pace of urbanization is fastest in Africa and South-East Asia, the exposure to nature in these populations is expected to fall dramatically [8].

## Data Availability

Not applicable.

## Abbreviations

ATS: American Thoracic Society
COPD: chronic obstructive pulmonary disease
CRDs: chronic respiratory diseases
CRS: chronic rhinosinusitis
ERS: European Respiratory Society
EUFOREA: european forum for research and education in allergy and airway diseases
EU: European Union
FCTC: Framework Convention on Tobacco Control
GARD: Global Alliance Against Chronic Respiratory Diseases
GSDR: Global Sustainable Development Report
IPBES: Intergovernmental Science-Policy Platform on Biodiversity and Ecosystem Services
MPOWER: monitor tobacco use and prevention policies, protect people from tobacco smoke, offer help to quit tobacco use, warn about the dangers of tobacco, enforce bans on tobacco advertising, promotion and sponsorship, raise taxes on tobacco
NCDs: non-communicable diseases
SDGs: sustainable development goals
SITRA: Suomen itsenäisyyden juhlarahasto: Finnish Innovation Fund
UN: United Nations
WHO: World Health Organization
